# Prevalence of hyperglycemia in pregnancy and influence of body fat on development of hyperglycemia in pregnancy among pregnant women in urban areas of Arusha region, Tanzania

**DOI:** 10.1186/s12884-019-2463-8

**Published:** 2019-08-28

**Authors:** Safiness Simon Msollo, Haikael David Martin, Akwilina Wendelin Mwanri, Pammla Petrucka

**Affiliations:** 1 Department of Food Biotechnology and Nutritional Sciences in School of Life Sciences, Nelson Mandela African Institution of Science and Technology, P. O. Box 477, Arusha, Tanzania; 2 Department of Food Technology, Nutrition and Consumer Sciences, Sokoine University of Agriculture, Morogoro, Tanzania; 3 College of Nursing, University of Saskatchewan, Saskatoon, Canada

**Keywords:** Hyperglycemia in pregnancy, Body fat, Risk factors, Arusha, Tanzania

## Abstract

**Background:**

Hyperglycemia in pregnancy is a medical condition resulting from either pre-existing diabetes or insulin resistance developed during pregnancy. This study aimed to determine the prevalence of hyperglycemia in pregnancy and influence of body fat percentage and other determinants on developing hyperglycemia in pregnancy among women in Arusha District, Tanzania.

**Methods:**

A cross–sectional study was conducted between March and December 2018 at selected health facilities in Arusha District involving 468 pregnant women who were not known to have diabetes before pregnancy. Blood glucose was tested by Gluco-Plus™ using the World Health Organization criteria at fasting and 2 h after consuming 75 g of glucose dissolved in 300 ml of water. Body fat was measured using a bioelectric impedance analyzer, mid-upper arm circumference using a regulated tape, weight using SECA™, blood pressure using a GT-868UF Geratherm™ machine, and height using a stadiometer. Demographic and maternal characteristics were collected through face to face interviews using a structured questionnaire.

**Results:**

The participants’ mean age was 28 years (SD ± 6), mid-upper arm circumference 27 cm (SD ± 3.7), body fat 33.72% (SD ± 7.2) and pre-pregnancy body mass index 25.6 kg/m^2^ (SD ± 5.5). One-third of participants had mid-upper arm circumferences ≥28 cm with 25% being overweight and 22.7% obese before pregnancy. Prevalence of hyperglycemia in pregnancy was 16.2% (*n* = 76) of which 13% had gestational diabetes and 3.2% diabetes in pregnancy. Hyperglycemia in pregnancy was significantly associated with body fat percentage (AOR 1.33; 95% CI: 1.22–1.44), family history of Type 2 diabetes mellitus (AOR 6.95, 95% CI: 3.11–15.55), previous delivery of babies ≥4 kg (AOR 2.3, 95% CI: 1.00–5.28), mid-upper arm circumference ≥ 28 cm (AOR 1.2, 95% CI: 1.09–1.32), and Type 2 diabetes mellitus symptoms (AOR 2.83, 95% CI: 1.53–6.92).

**Conclusion:**

The prevalence of hyperglycemia in pregnancy was high, particularly among women with history of delivering ≥4-kg babies, increased body fat, mid-upper arm circumference, symptoms and/or family history of Type 2 diabetes mellitus. These findings identify opportunities to further explore the utility of body fat percentage and other determinants for rapid screening and management of hyperglycemia in pregnancy.

## Background

Hyperglycemia in pregnancy (HIP) is one of the most common pregnancy-specific health challenges [1]. Hyperglycemia first detected at any time during pregnancy should be classified either as diabetes mellitus in pregnancy (DIP) or gestational diabetes mellitus (GDM)**.** Hence, HIP is a result of either pre-existing diabetes or insulin resistance developed during pregnancy, a condition known as gestational diabetes mellitus (GDM) which is defined as impaired glucose tolerance first recognized during pregnancy [2–4]. This condition occurs due to pregnancy-induced changes in maternal glucose metabolism and insulin sensitivity, whereby demand for insulin production on the mother’s pancreas increases as the pregnancy proceeds [5]. In most instances, these women can meet the increased insulin demand, but failure to accommodate results in poor glycemic control. This condition may disappear spontaneously after delivery, but, if misdiagnosed and mismanaged, may lead to persistent long-term health risks to the mother and the child, such as predisposition to obesity and development of Type 2 diabetes mellitus (T2DM) within five to 10 years postpartum [6].

Globally, the prevalence of GDM among women aged 20–49 years was estimated to be 17% affecting 21.4 million live births with more than 90% cases occurring in low- and middle-income countries [6]. Estimates suggest that GDM ranges between 6 and 18% in East and West Africa with pockets of higher prevalence (i.e., 19.5% in urban areas of Tanzania) [7, 8]. Inaction on the HIP agenda will lead to future high prevalence and contribute to an increased disease burden on the health system. Considering the disproportionate rates of overweight/obesity among Tanzanian pre-pregnant women in urban (42%) and rural (21%) locations, localized considerations are required [9]. Women with hyperglycemia are at high risk of hypertension, abortion/miscarriage, and/or a pregnancy resulting in a newborn who is large for gestational age (macrosomia), preterm birth, and/or incur perinatal death [10]. A number of risk factors contributing to HIP include family history of DM, previous delivery to macrosomia or stillbirth, intrauterine fetal death (IUFD), maternal obesity, and preterm delivery [11].

In addition to sedentary lifestyle, maternal height, dietary factors, cigarette smoking, and extreme pregnancy weight gain accompanied by high body fat accumulation place women at risk of HIP. Body fat percentage can alter body composition leading to pregnancy diabetes and other complications, such as pregnancy induced hypertension and predisposition of the newborn to overweight later in life [12–14]. It can also affect growth of the fetus; therefore, assessment of change in body fat content is important as predictive of both maternal and child health [15]. Body mass index (BMI) is a commonly used standard measure for assessing body fatness [16]; however, it does not distinguish between fat and lean body mass [17]. The delay of onset of antenatal care (ANC) means most women do not know their pre-pregnancy weight which makes it difficult to estimate BMI and weight gain during pregnancy which is reported to be strongly correlated with fat mass change [18]. This pre-pregnancy metric is even more important because, in addition to body fat and lean body mass, the fetal mass and amniotic fluid constitute an unknown contribution to total body mass of the mother which is indistinguishable within the BMI calculation. Furthermore, women, who are not obese by traditional weight criteria, may have an increased percentage of body fat distributed predominantly in the abdominal region, which leads to increased risk of HIP [19]. Studies done with T2DM patients have identified that fat deposits within skeletal muscle and liver cells is a major contributing factor to insulin resistance; however, there is lack of evidence on whether fat deposition during pregnancy adequately explains the acquisition of insulin resistance as a marker for HIP [20, 21].

Few studies have assessed the effects of body fat on development of GDM with most extant studies considering Caucasian and Asian populations [22, 23]. This variance requires redress as risks for HIP differ across ethnic, geographic, and genetic lines due to differences in body composition, lifestyles, genetic susceptibility, as well as healthcare system capacities [24]. Some populations may have lower BMI but high rates of HIP. For example, Asian populations tend to have a lower BMI, but accumulate visceral fat and develop abdominal obesity [25] which is positively associated with insulin resistance and impaired β cell function [26, 27].

In addition to body fat percentage, MUAC has a strong correlation with BMI [16] therefore it can be used instead of BMI as a much simpler, cheaper anthropometric measurement, which does not change significantly during pregnancy. Hence, it may be a better indicator of pregnancy body fat and nutrition status than BMI [28]. MUAC does not need mathematical calculations, additional equipment, and regular equipment standardization which are important considerations in limited resource settings [29]. Furthermore, MUAC is highly associated with GDM [30] which means that a woman with a MUAC beyond the normal value is at increased risk of GDM compared to the one with normal MUAC value.

The current study was conducted in Arusha District, which has a known high prevalence of T2DM (16.2%) especially in urban (22.9%) compared to rural (9.9%) areas [31], although this variance may, in part, reflect undiagnosed and unmanaged HIP outside the urban centres. This situation led to the need to determine the prevalence of HIP and its associated risk factors especially the less explored body fat percentage for possible interventions to prevent short- and long-term adverse effects to the mother and her newborn.

## Methods

A cross-sectional study was conducted in urban areas of Arusha District between March and December of 2018. The study involved pregnant women attending antenatal clinics at Ngarenaro and Kaloleni Health Centers in Arusha District. The two centers were purposively selected due to their central location and large numbers of pregnant women (on average 40–100 per day) accessing Ante-Natal Care (ANC) services from across the District, thereby representing the demographics of this predominantly urban patient population. The study included women with gestational ages between 24 and 36 weeks anticipating delivery at one of the participating centers. Pregnant women with previously diagnosed diabetes and selected conditions, such as sickle-cell anemia or cancer, were excluded from the study. The aim, procedure, benefits, and negative effects of the study were explained to all enrolled women agreeing to participate and signed informed consents. The study was approved by the Tanzania National Institute of Medical Research (NIMR) with a reference number NIMR/HQ/R.8a/Vol.IX/2694.

The eligible women were selected with assistance of the nurse in-charge resulting in a total of 468 women being involved in the study. Sample size was determined in accordance with the formula for prevalence studies [32]. Due to limited large-scale data on the prevalence of HIP in Tanzania, 50% was used as prevalence in the formula for maximum reality [33] with an assumed attrition rate of 20%. Random sampling using a table of numbers was used to select women to participate in the study and due to the high number of pregnant women attending ANC in the areas, 12 women were assessed per day. All participants (100%, *n* = 468) completed fasting blood glucose tests and face-to-face interviews using structured questionnaires. In addition, nearly all (97.8%, *n* = 446) of the participating women completed Oral Glucose Tolerance Test (OGTT) procedures.

### Assessment of demographic characteristics and selected risk factors for HIP

Recalled information respecting weight before pregnancy, first generation family history of T2DM, previous history of GDM, symptoms of T2DM, and previous delivery of babies weighing ≥4 kg at birth were collected through face-to-face interviews using a structured questionnaire. Other maternal characteristics, such as age, perinatal and prenatal death, gravidity, education level, occupation, marital status, and weight during the first pre-natal visit, were obtained from the participants’ ANC records.

### Laboratory tests for HIP

Women were required to fast overnight before capillary blood was taken using a finger prick with a sterile lancet after cleaning the site with an antiseptic alcohol swab. Fasting blood glucose and 2 h OGTT following the consumption of anhydrous glucose of 75 g dissolved in 300 ml of water [3, 34] were measured using Gluco-plus™ (Glucoplus Inc. 2323 Halpern, Ville St. Laurent, Quebec, Canada). The capillary plasma glucose values obtained were converted into venous plasma glucose using the regression equation developed for diabetes screening in the low resource areas where venous blood is challenging [35]. Women whose fasting blood glucose levels were ≥ 5.1 mmol/L were requested to return the next day for another fasting glucose test. Women were classified as having HIP if they met the criteria for DIP and GDM which is fasting plasma glucose (5.1–6.9 mmol/l (92–125 mg/dl), or a 2-h plasma glucose (8.5–11.0 mmol/l (153–199 mg/dl) following a 75-g oral glucose load. The 1-h OGTT was not considered because there are no established criteria for the diagnosis of diabetes based on the 1-h post-load value and one reading is enough. Hence a single blood draw after 2 h is more reasonable than after every 1 hour [3]. In addition, DIP was classified by fasting plasma glucose ≥7.0 mmol/l (126 mg/ dl) and/or 2-h plasma glucose ≥11.1 mmol/l (200 mg/dl) following a 75 g oral glucose load [34]. Furthermore, all women identified with HIP were referred to see the doctor for further actions.

### Anthropometric assessments

Mid-upper arm circumference (MUAC) was measured using a non-stretchable tape and women were categorized as normal with a MUAC of < 28 cm and overweight with MUAC ≥28 cm. Mid upper arm circumference was used to supplement BMI due to its relative stability during the course of pregnancy and high correlation with pre-pregnancy BMI, making it a better indicator of pre-pregnancy body fat and nutrition status than the BMI [16, 36]. Weight was measured with minimal clothing and without shoes using a digital bathroom weighing scale (SECA-Germany), placed on a flat surface and recorded to the nearest 0.1 kg. Body fat percent was determined using a bioelectric impedance analyzer (Tanita TBF 105 Fat Analyzer™) which includes adjustment for age, height, and sex. The body fat percentage was treated as a continuous variable and there are no established classification criteria for pregnancy. Blood pressure was measured using a GT-868UF Geratherm™ machine and women were classified using the blood pressure categories of systolic 130 to 139 mmHg or diastolic 80 to 89 mmHg for Stage 1 hypertension and systolic ≥140 mmHg or diastolic ≥90 mmHg for Stage 2 hypertension [37].

### Data analysis

Data collected were analyzed using the Statistical Package for Social Science™ (SPSS) Version 20 and descriptive statistics, such as frequency, mean and percentage, were obtained. Chi-square test was used for comparing the selected categorical variables between women with and those without HIP. Blood glucose levels were dichotomized, and univariate analysis was done for the variables associated with HIP to obtain crude odd ratios. Multiple logistic regression analysis explored whether different factors had significant association with HIP where the quantitative variables such as body fat percent, BMI and MUAC were treated as continuous variables. Statistical inference was based on 95% confidence intervals (CIs) and significance at *p* value ≤0.05. Some factors were potential confounders, hence they were introduced into the model during the analysis, such as age, and gestational age. The variables with missing data (i.e., BMI) were analyzed by “pairwise” deletion where the statistical procedure used cases that contain some missing data. Hence, the entire case/respondent was not left out of subsequent analyses for elements on which data were complete. Other variables, which were missing due to mistakes in recording, were traced back from the participants as their phone numbers and other contacts were recorded. Other missing data were traced from ANC records available in the health center. Student’s t-test was used to compare the means for HIP and non-HIP groups for continuous variables, such as mean OGTT and fasting glucose values.

## Results

A total of 468 pregnant women participated in the study at Ngarenaro and Kaloleni Health Centers in Arusha, Tanzania. They were all black African by ethnicity. Most were married (95.9%, *n* = 459), and over half had attended primary school (58.8%, *n* = 275) and were self-employed (55.8%, *n* = 261) primarily in small businesses earning an average income of < 250,000 Tanzanian Shillings (TSH) per month (approximately 110 American dollars). The mean age of the women was 28 years (SD ± 5.84), of which 65.6% were ≥ 25 years old. The mean gestational age at the start of ANC was 18 weeks (SD ± 5.62) and 28.5 weeks (SD ± 3.82) at the beginning of this study where nearly two-thirds (62.2%, *n* = 354) were beyond 28 weeks gestation at time of entry to the study with 50.4% (*n* = 236) reported as second or third gravidity (Table 1).

**Table 1:** Demographic and selected maternal characteristics of pregnant women

Respondent Variables	Frequency (n)	Percent (%)	Mean (±SD)
Education levels			
Never went to school	8	1.7	
Primary level	275	58.8	
Secondary level	164	35.0	
College/University	21	4.5	
Marital status			
Single	16	3.4	
Married or Cohabiting	439	93.8	
Divorced/Separated	13	2.8	
Occupational status			
Formally employed	46	9.8	
Self employed	261	55.8	
Unemployed	161	34.4	
Income per month (TSH)			
<250,000	255	54.5	
250,000-450,000	33	7.1	
≥500,000	13	2.8	
I don’t know	167	35.7	
Age			
<25 years	164	35.0	28 (SD ± 5.84)
≥25 years	304	65.0	
Gestational age at first visit			
<12 weeks	57	12.2	
12–24 weeks	363	77.6	18 (SD ± 5.62)
25–36 weeks	48	10.2	
Gestational age at study commencement			
24–28 weeks	291	62.2	28 (SD ± 3.82)
>28 weeks	117	37.8	
Gravidity			
Prime	142	30.3	
Second and third	236	50.4	3 (SD ± 1.20)
Fourth and above	90	19.2	

The mean self-reported pre-gestational weight was 67 kg (SD ± 12.5). This weight was used to determine pre-pregnancy BMI of the women in which 25.2% (*n* = 60) were classified as overweight and 22.7% (*n* = 54) as obese. The measured mean height was 159 cm (SD ± 6.3), body fat 33.7% (SD ± 7.2), and MUAC 27 cm (SD ± 3.8) in which 36.1% (*n* = 164) had MUAC ≥28 cm (Table 2).

**Table 2:** Anthropometric measurements of the pregnant women

Variables tested	Frequency (n)	Percent (%)	Mean (SD)
BMI (kg/m^2)^ from self-reported pre-pregnancy weight			
Underweight (< 18.5)	15	6.3	
Normal (18.5–24.9)	109	45.8	25.5 (SD ± 6.3)
Overweight (25–29.9)	57	25.2	
Obese (≥30)	48	22.7	
MUAC			
<28 cm = Normal	299	63.9	27 (SD ± 3.8)
≥28 cm = Above normal	169	36.1	
Body fat percentage			33.4 (SD ± 7.8)
Self-reported pre-pregnancy weight (kg)			67 (SD ± 12.5)
Height (cm)			159 (SD ± 6.3)

All pregnant women who participated in the study (100%, *n* = 468) completed fasting blood glucose tests and 97.8% (*n* = 446) underwent an OGTT. The prevalence of HIP was 16.2% (95% CI: 13–19.9) of which 3.2% (*n* = 15) had DIP and 13% (*n* = 61) GDM using the WHO [3] criteria. Among the women assessed, 10.9% (*n* = 51) reported to have symptoms of T2DM, such as extreme tiredness, diaphoresis (excessive sweating), and polydipsia (excessive thirst) (Table 3).

**Table 3:** Laboratory tests for glucose and protein among pregnant women

Variables Tested	Frequency(n)	Percent (%)
Pregnancy glycemia status		
Normal	392	83.8
HIP	76	16.2
GDM	61	13.0
DIP	15	3.2
Having any symptoms of diabetes		
Yes	51	10.9
No	417	89.1

The overall mean of fasting blood glucose was 4.5 mmol/L (SD ± 1.3), with the HIP sub-group having a mean of 6.4 mmol/L (SD ± 1.5), yielding a significantly higher (*p* < 0.001) mean compared to the non-HIP group (4.2 mmol/L, SD ± 0.9). The overall mean for OGTT was 5.5 mmol/L (SD ± 1.06) and was significantly higher (*p* < 0.001) among the HIP (8.3 mmol/L, SD ± 1.3) compared to the non-HIP group (5.5 mmol/L, SD ± 0.1) (Table 4).

**Table 4:** Mean blood glucose comparisons between women with and without HIP

Variables Tested	Frequency(n)	Mean	SD	*P*-value
Fasting blood glucose				
General fasting blood glucose	468	4.5	±1.3	
Normal (< 5.1 mmol/L)	397	4.2	±0.9	0.000*
HIP (≥5.1 mmol/L)	71	6.4	±1.5	
OGTT				
General OGTT values	446	5.6	±1.06	
Normal (< 8.5 mmol/L)	436	5.5	±0.1	0.000*
Glucose intolerance (≥8.5 mmol/L)	10	8.3	±1.3	

*significant

The prevalence of HIP was significantly higher (*p* < 0.000) among women with a family history of T2DM (44.9% vs 10.5%, *p* < 0.001); MUAC ≥28 cm (23.1% vs 12.4%, *p* = 0.003); previous delivery of an infant ≥4 kg (47.1% vs 4.4%, *p* < 0.001); and symptoms of T2DM (49.1% vs 12.2%, *p* < 0.001). The observed prevalence of HIP did not differ significantly among women with histories of prenatal or perinatal death, high blood pressure, high maternal age, or smoking and alcohol intake habits (*p* > 0.05) (Table 5).

**Table 5:** Comparison of selected characteristics between women with and without HIP

Variable	With HIP	Without HIP	*P*-value
MUAC			
<28 cm (Normal)	37 (12.4%)	262 (87.7%)	0.003*
≥28 cm (overweight/obese)	39 (23.1%)	130 (76.9%)	
BMI pre-pregnancy			
Underweight and Normal	19 (15.3%)	105 (84.7%)	0.251
Overweight and Obese	24 (21.1%)	90 (78.9%)	
Family history of diabetes			
Yes	35 (44.9%)	43 (55.1%)	0.000*
No	41 (10.5%)	349 (89.5%)	
Previous delivery to ≥4 kg baby			
Yes	48 (47.1%)	54 (52.9%)	0.000*
No	10 (4.4%)	219 (95.6%)	
Mother’s maternal age			
<25 years	24 (14.4%)	143 (85.6%)	0.414
≥25 years	52 (17.3%)	249 (82.7%)	
Symptoms of T2DM			
Yes	25 (49.0%)	26 (51.0%)	
No	51 (12.2%)	366 (87.8%)	0.000*
Prenatal death			
Yes	10 (15.9%)	53 (784.1%)	0.467
No	48 (17.9%)	220 (82.1%)	
Perinatal death			
Yes	1 (10.0%)	9 (90.0%)	0.538
No	29 (20.1%)	115 (79.9%)	
Alcohol intake			
Yes	6 (19.4%)	25 (80.6%)	0.665
Never	63 (15.5%)	344 (84.5%)	
Stopped during pregnancy	4 (25.0%)	12 (75.0%)	
Smoking			
Never	76 (16.2%)	390 (83.7%)	0.533
Stopped during pregnancy	0 (0.0%)	2 (100.0%)	

*Significant

The selected risk factors were analyzed using multiple logistic regression analysis to determine their association with HIP. A significant association was observed with increased body fat percentage (AOR 1.33, 95% CI: 1.22–1.44), family history of T2DM (AOR 6.95, 95% CI: 3.11–15.55) and previous delivery of babies ≥4 kg (AOR 2.3, 95% CI: 1.00–5.28), and/or having any symptoms of T2DM (AOR 2.83, 95% CI: 1.53–6.92) in the first model. The second model replaced body fat percentage with MUAC and the association remained consistently significant in all factors of the first model with addition of MUAC (AOR 1.2, 95% CI: 1.09–1.32) (Table 6).

**Table 6:** Odd Ratios of select risk factors for hyperglycemia in pregnancy

Univariate analysis				
Risk factors	Crude OR (95%CI)		*P*-value	
Body fat percentage	1.29 (1.21–1.36)		0.000*	
MUAC	1.16 (1.09–1.24)		0.000*	
BMI	1.01 (0.99–1.03)		0.294	
Symptoms of T2DM				
No	1			
Yes	6.9 (3.7–12.86)		0.000*	
Family history of T2DM				
No	1			
Yes	6.93 (3.99–12.02)		0.000*	
Previous delivery of ≥4 kg baby				
No	1			
Yes	5.9 (3.13–11.03)		0.000*	
Preterm delivery				
No	1			
Yes	1.2 (0.366–3.826)		0.779	
Multivariate analysis				
Model 1		Model 2		
Risk factors	Adjusted OR (95%CI)		Adjusted OR (95%CI)	*P*-value
Body fat percentage	1.33(CI:1.22–1.44)	0.000*	NA	
MUAC	NA		1.2 (CI: 1.09–1.32)	0.000*
Symptoms of T2DM				
No	1		1	
Yes	2.83(CI: 1.53–6.92)	0.023*	3.66(CI: 1.64–8.18)	0.002*
Family history of T2DM				
No	1		1	
Yes	6.95(CI: 3.11–15.55)	0.000*	6.04(CI: 3.04–12.01)	0.000*
Previous delivery to ≥4 kg babies				
No	1		1	
Yes	2.3(CI: 1.00–5.28)	0.049*	3.5(CI: 1.71–7.36)	0.001*

The univariate analysis also included periterm death, maternal age, gestational age, and blood pressure with no significant association with HIP. Multivariate analysis included family history of Type 2 diabetes mellitus (T2DM), previous delivery of ≥4 kg babies, body fat percentage and MUAC in which body fat was replaced by MUAC in model 2.The abbreviation NA means not applicable in the particular model

## Discussion

The current study was carried out to determine the prevalence of HIP and influence of body fat percent and other risk factors on development of HIP among women in Arusha District. The overall prevalence of HIP was found to be 16.2%, of which 3.2% had DIP and 13% GDM according to WHO criteria [3]. Gestational diabetes mellitus is not the only form of hyperglycemia which may first be detected during pregnancy as DIP is a more severe form of HIP in which diagnostic glucose levels are the same as those of non-pregnant adults [3]. Therefore, the prevalence of hyperglycemia in pregnancy in this study combined GDM and DIP as it is important to include pregnant women with pre-existing diabetes in designing interventions. Moreover, DIP increases the vulnerability to complications because of the degree of hyperglycemia and the uncertainty as to whether the onset of hyperglycemia was prior to pregnancy or it developed during pregnancy [2]. This study provides evidence for designing interventions like pre-pregnancy testing of blood glucose levels, emphasis on health education to improve health seeking behaviors among women of reproductive age. In Tanzania, due to limited resources, most women are not tested for glucose levels before pregnancy. It is therefore important that HIP is considered to ensure thorough glucose control before conception, throughout pregnancy and postpartum for prevention of diabetes in progressive pregnancies. Hence, including DIP and GDM together may strengthen the approach and allow recognition of general prevalence of hyperglycemia to open dialogue on future GDM approaches that align with global standards for all women in Tanzania. The observed prevalence of HIP may increase burden to the health system if no immediate actions are taken. The need exists to explore the associated modifiable risk factors including body fat to enhance self-care practices and prevent poor pregnancy outcomes as well as T2DM later in life. A similar study conducted in Tanzania using the same WHO [3] criteria reported that the prevalence of GDM was 19.5 and 3% had DIP in Moshi Kilimanjaro Region which is higher than the current study [8]. The differences in prevalence of hyperglycemia observed may be due to nature of the diets, cultural differences in food preparation, and care during pregnancy which needs further exploration. In addition, another similar study done in India supported our findings on the consideration of HIP in screening hyperglycemia during pregnancy and reported that, HIP was prevalent in 18.9% of the study population where by 16.3% had GDM and 2.6% DIP [38].

The prevalence of HIP in the present study was significantly associated with increased body fat percentage, family history of T2DM, previous delivery of ≥4 kg babies, and reported symptoms of T2DM. When body fat percentage was replaced by MUAC in the second model, increased in MUAC was independently associated with HIP. On other hand, BMI obtained from the recalled pre-pregnancy weight was not significantly associated with HIP even after been replaced for MUAC and/or body fat percentage in the models, making it a weak determinant of HIP. In this case, body fat percentage together with MUAC can be used instead of BMI as determinants of HIP due to their independent association with HIP. Moreover, the majority of the women in the current study could not recall their pre-pregnancy weight making it difficult to estimate their BMI. This finding is supported by Mwanri et al. [30] who found that BMI could not be estimated for most of the women, as they could not recall their pre-pregnancy weight. Another study reported that less than half of the mothers could recall their pre-gestational weight [39].

Although pre-gestational weight can be estimated with a recorded weight within 15 weeks of pregnancy [39], women in the current study started ANC with a mean gestational age of 18 weeks; hence, their pre-pregnancy weights were indeterminate. A similar study reported that, since most of the women appeared late to the ANC with a mean gestational age of 20 weeks, it was difficult to obtain pre-gestational weight [36]. Hence, information on changes in body fat content is required due to its independent association with HIP which is further supported in a previous report that the risk of GDM was independently associated with high body fat percent, similar to the findings in people with T2DM [23]. With these associations, it would be important to utilize these simple factors to identify pregnant women at risk for HIP so that prevention measures, such as lifestyle modifications, can be implemented to prevent poor pregnancy outcomes [40].

Women with a history of delivering babies ≥4 kg at birth were at twice the risk of GDM compared to their counterparts even after adjusting for body fat percentage, pre-pregnancy BMI, gestational age, and MUAC. This finding reveals how maternal health status is the determinant of health of the newborn. Another study found that a previous history of delivering a baby weighing ≥4 kg was an independent predictor of developing GDM [41]. This finding means that a woman with history of previous pregnancy, which resulted in a child with a high birth weight, is at an increased risk of GDM in progressive pregnancies [42].

The current study also reported that women with family history of diabetes were at almost 7 times greater risk of HIP compared to their non-affected counterparts meaning that HIP can be influenced by genetic predisposition and/or lifestyle practices, such as dietary and low physical activities. Another study showed that GDM is considered to result from interaction between genetic and environmental risk factors [43]. Pregnancy triggers a series of metabolic imbalances that lead to a diabetic state in women who are already genetically predisposed to develop diabetes [44]. Of note, GDM and T2DM share a similar genetic background [45] which might be a reason to why women with strong first-degree family history of T2DM are at high risk of HIP. Hence, genetic predispositions to T2DM or HIP should not be ignored. Other studies concur with the current report that family history of diabetes remained significantly associated with GDM even after adjustment for other co-variates [37, 46].

Nevertheless, some women with HIP reported having one or more symptoms of T2DM, such as extreme tiredness, diaphoresis (excessive sweating), and polydipsia (excessive thirst) and increased frequent urination. These symptoms were found to have a strong independent association with HIP even after adjusting for MUAC, BMI, gestational age and family history of T2DM. Considering the asymptomatic nature of HIP [19], these women may have pre-existing T2DM which was not diagnosed before pregnancy because, 3.2% (*n* = 15) of the women were found to have high cut off points which indicated T2DM. This creates a need to promote pre-pregnancy preparation that include regular screening for diabetes before conception for earlier efforts to prevent the development of HIP. Hyperglycemia in pregnancy has few symptoms and should be diagnosed by screening during pregnancy [47]. Another study reported that overt symptoms of HIP are rare and may be difficult to distinguish from normal pregnancy symptoms creating a need for confirmatory OGTT [19]. Hyperglycemia in pregnancy can be influenced by first generation family history of T2DM and, when undiagnosed and unmanaged, can lead into recurrent HIP in subsequent pregnancies and/or T2DM later in life [6, 46]. Hence, symptom clusters with other risk factors can be used for identification of women who need screening for HIP especially in low- and middle-income countries where universal screening is not possible.

### Limitation of the study

Some data were collected based on participants’ ability to recall. For example, BMI was based on recall data where almost half of the respondents could not report their pre-pregnancy weight leading to missing data; hence, interpretations on the rate of overweight and obesity need to be done carefully. In addition, GDM was tested by one out of two elevated glucose levels instead of one out of three values as recommended [3].

### Conclusions and recommendations

The prevalence of HIP was observed to be high in urban areas of Arusha District and significantly associated with family history of T2DM, increased MUAC, body fat percentage, and having one or more symptoms of T2DM. The reported risk factors can be used to identify women at risk of HIP early enough for earlier interventions to be taken to prevent poor pregnancy outcomes especially in resource poor settings where universal screening is challenging. Modifiable risk factors, like body fat percentages for assessing nutrition status in pregnancy, need to be further explored to identify the proper methods for body fat estimation and develop criteria for classification in pregnancy. Prevention strategies for HIP need to be directed towards the knowledge and control of the risk factors reported in this study and others for promoting self-care before, during, and after pregnancy. This proactive approach can potentially reduce the impacts of non-modifiable risk factors, such as age and genetic predisposition in developing HIP.

## Data Availability

The datasets used and analyzed during the current study will be available on reasonable request to the corresponding author. This is because the data set contains other data that have not yet been analyzed as the study involves a prospective cohort part which is still going on in the area.

## Abbreviations

ANC: Antenatal care
AOR: Adjusted Odd Ratios
BMI: Body Mass Index
CI: Confidence Interval
GDM: Gestational Diabetes Mellitus
HIP: Hyperglycemia in Pregnancy
MUAC: Mid-Upper Arm Circumferences
OR: Odd Ratio
T2DM: Type 2 Diabetes Mellitus
